# Spectral Signatures of the Developmental Stages of *Sphenophorus levis* (Vaurie, 1978) (Coleoptera: Curculionidae) on a Natural Diet

**DOI:** 10.3390/insects17050465

**Published:** 2026-04-30

**Authors:** Pedro Gomes Peixoto, Gabriela Maria Martins Ferreira, David Luciano Rosalen, Souradji Idrissou Bachirou, Sergio Antonio De Bortoli

**Affiliations:** 1 Department of Crop Protection, School of Agricultural and Veterinary Sciences, São Paulo State University (UNESP), Via de Acesso Professor Paulo Donato Castelane Castellane S/N-Vila Industrial, Jaboticabal 14884-900, SP, Brazil; sergio.bortoli@unesp.br; 2 Department of Rural Engineering, School of Agricultural and Veterinary Sciences, São Paulo State University (UNESP), Via de Acesso Professor Paulo Donato Castelane Castellane S/N-Vila Industrial, Jaboticabal 14884-900, SP, Brazil; gmm.ferreira@unesp.br (G.M.M.F.); david.rosalen@unesp.br (D.L.R.); ib.souradji@unesp.br (S.I.B.)

**Keywords:** spectral library, insect biology, sugarcane weevil, light ecology, spectroradiometry, plant health, integrated pest management, sugarcane weevil, hyperspectral imaging, sexing

## Abstract

This study investigated the spectral profiles of different developmental stages of *Sphenophorus levis*, possibly the most important sugarcane pest in the Southeast, Midwest, and South regions of Brazil, given its economic relevance, distribution, and management challenges in sugarcane plantations. Hyperspectral remote sensing (HRS) techniques were employed. Spectral signatures were obtained for eggs, larvae, pupae, and adults using a Pika-L sensor (range, 400–1000 nm). This made it possible to identify distinct spectral patterns for each developmental stage, allowing for the differentiation between eggs, larvae, pupae, and adults. The results indicate the presence of such distinct spectral patterns, which may suggest the potential of hyperspectral imaging for the non-destructive identification of each stage of *S. levis*, although further investigations are needed to validate this approach. The eggs showed high variability in reflectance, possibly related to compositional changes during embryonic development. The larval instars exhibited a decrease in reflectance with age, likely due to cuticular changes. The evaluated pupae presented spectral differences that coincidentally enabled early sex determination, while adult males and females can also be distinguished based on their reflectance profiles, with females typically showing higher overall reflectance. This spectral library provides a foundation for the determination and sexing of instars, as well as the assessment of quality patterns in this important sugarcane pest. This study highlights the integration of advanced hyperspectral imaging technologies with insect biology to enhance integrated pest management strategies and sheds light on the biological and ecological aspects of the species.

## Simple Summary

This study investigated the spectral signatures of different developmental stages of *Sphenophorus levis*, a pest of great importance in sugarcane. The reflectance of eggs, larvae, pupae, and adults was analyzed using hyperspectral remote sensing (HRS) techniques. The results showed distinct spectral patterns that allowed for the non-destructive identification of each stage. The eggs exhibited high variability in reflectance related to changes in their composition, whereas the larvae showed a decreasing reflectance pattern with age. It was also possible to differentiate between male and female adults based on reflectance, with females displaying higher reflectance. These findings may be valuable for integrated pest management, providing a foundation for the effective monitoring and management of *S. levis* and contributing to the ecological and biological understanding of this pest. This study highlights the importance of integrating advanced technologies with insect biology to improve management strategies.

## 1. Introduction

Brazil is the world leader in sugarcane production [1] (*Saccharum* spp. Magnoliopsida: Poaceae [2]). This leadership is maintained even though production indicators point to a decrease, with production expected to reach 678.67 million tons (2024/25 harvest), a reduction of 4.8% compared to the previous harvest. Adverse climatic effects, such as low rainfall and high temperatures, have been identified as the main factors behind the drop in productivity for the period [1], along with an intensification of pest insect attacks.

Sugarcane is subjected to consistent and permanent pest attacks, suffering multiple infestations in all parts of the plant and at every stage of growth and development [3]. A myriad of insect pests can be found in sugarcane fields, the main ones being *Diatraea saccharalis* (Fabricius, 1794) (Lepidoptera: Crambidae), *Mahanarva* spp. (Stål, 1854) (Hemiptera: Cercopidae), *Saccharicoccus sacchari* (Cockerell, 1895) (Hemiptera: Pseudococcidae), and *Migdolus fryanus* (Westwood, 1893) (Coleoptera: Vesperidae) [3]. However, pests of secondary importance have been drawing increasing attention, particularly soil-dwelling pests such as *Hyponeuma taltula* (Schaus, 1904) (Lep.: Erebidae) [4] and *Sphenophorus levis* (Vaurie, 1893) (Col.: Curculionidae) [5].

*S. levis* has been reported as a sugarcane pest since 1977 [5], with consistent attacks on the root structure of sugarcane, called the rhizome, and sometimes even reaching the first basal internode [5,6]. These damages lead to massive productivity losses (~30%) in infested cane fields [5,6,7], as well as high tiller mortality rates (~60%) and reductions in plantation longevity [6]. The values obtained for the economic damage threshold (EDT) indicated that EDTs were 5.93% and 4.85% for rainfed and irrigated crops, respectively, under chemical control, whereas for biological control, the values were 4.15% and 3.40% [7], respectively, using the “toco-atacado” methodology. The “stump-to-furrow sampling method” refers to the Brazilian field technique known as “toco-atacado”, in which the soil is excavated from the sugarcane stump (“toco”) toward the furrow (“atacado”) to inspect the root zone for soil-dwelling pests. The results obtained with this method are typically expressed as the percentage of infestation [8]. Thus, this pest, previously considered secondary, has been drawing attention and demanding efforts from producers and researchers to deepen their understanding and develop management techniques.

Adults exhibit peaks of activity during the night and generally remain below ground level, under straw, or between the shoots at the base of sugarcane clumps (rhizomes) [6,9]. With gregarious habits, males emit compounds that attract females and other males [10,11]. Additionally, they are long-lived organisms with a lifespan of up to 200 days [12], which allows them to initiate the colonization of host plants. After mating, females lay eggs at the base of the rhizome, inside the rhizome tissues [12]. Females have a high biotic potential (~70 eggs/female); the eggs develop within 7–12 days after being laid, and approximately 50 days later, the larvae begin the pupation process, which lasts approximately 12 days, protected inside the rhizome from which the adults emerge [12]. The total cycle is 250 days under ideal conditions.

It can be said with certainty that there is a lack of data and basic biological information (little scientific literature) that would allow for an understanding of the biology and ecology of *S. levis*. Given its significant importance as a pest in sugarcane fields and its economic risks, understanding its bioecological aspects is essential to support control strategies. Insect rearing is a pillar of Integrated Pest Management, responsible for providing biological data that can inform decision-making in the field. Consequently, studies aimed at developing spectral profiles using remote sensing (RS) have gained prominence.

This technique involves the application of sensors and understanding of luminous properties, especially transmittance, absorbance, and reflectance. Light is an essential electromagnetic radiation that displays different properties in a spectrum. The electromagnetic spectrum covers various ranges, including ultraviolet (UV), visible light, and infrared (IR) radiation. The visible spectrum, approximately 400–700 nanometers (nm), represents only a narrow segment of this range, where human vision is optimized [13]. Within the spectrum, violet is ~380–450 nm, blue is ~450–495 nm, green is ~495–570 nm, yellow is ~570–590 nm, orange is ~590–620 nm, and red is ~620–750 nm, with values above 750 nm and below 380 nm being invisible to the human eye [14]. The use of sensors expands human vision and enables the identification of patterns that are not detectable by human sight, given that the visible electromagnetic spectrum is limited [14,15].

However, this limitation restricts our ability to perceive beyond this range, leading to limitations whose implications are especially notable in areas such as agriculture, where the inability to detect information outside the visible spectrum can result in inaccurate human assessments [13,14,15]. Thus, the use of equipment for RS on a large scale can provide a comprehensive view of the distribution and intensity of infestations throughout the cultivated area, assisting in the strategic planning of Integrated Pest Management (IPM) [14]. It can also be applied in the context of insect biology studies [16,17,18], which may aid in pest control by enabling the precise identification, monitoring, and differentiation of life stages [19] and may be used as indicators of genetic changes in populations and egg infestations [20].

The applications of these techniques in agriculture primarily target the early detection of stresses (biotic and/or abiotic) [14]. This knowledge, combined with the principles of IPM, enables faster and more effective interventions and supports studies on the biology of pests and beneficial insects, an important pillar of IPM [14]. Spectral analysis can reveal subtle changes in the surface reflectance. The analysis of spectral signatures obtained by RS is a valuable tool. Therefore, by integrating biological data with spectral analyses, it is possible to optimize mass production, allowing for sexing, determination of quality standards, and identification of specific developmental stages.

This study aimed to investigate the spectral signatures of *S. levis* at different developmental stages. This proposal also seeks to explore the practical applications of this library, such as laboratory rearing, determination of biological parameters (specific to each instar), and sexing of individuals using the hyperspectral remote sensing (HRS) technique.

## 2. Materials and Methods

### 2.1. Experimental Area

A commercial sugarcane cultivation area is in a region with a climate classified as AW by the Köppen system [21] in the central part of the State of São Paulo, Brazil. In this area, we conducted periodic (monthly) collections to obtain healthy individuals under suitable conditions for experimentation. Collection is a harmful and stressful practice for *S. levis*, resulting in initial mortality that renders a large proportion of the collected organisms unsuitable for analysis.

### 2.2. Species Confirmation

Some individuals (adults) were taken to the laboratory, euthanized (hypothermia), mounted, and sent to a specialist for species identification and were cataloged at the National Institute of Coleoptera (INCol), under the responsibility of Prof. Dr. Fernando Z. Vaz-de-Mello.

### 2.3. Acclimatization and Maintenance in the Laboratory

The insects collected using bait cane (sugarcane billets cut lengthwise about 30 cm and placed on the ground, covered with sugarcane straw) were taken to the Laboratory of Insect Biology and Rearing (LBCI), where a 3% (*v*/*v*) copper sulfate solution was applied to remove possible contaminants. In particular, *Beauveria bassiana* (Balsamo-Crivelli) Vuillemin, an entomopathogenic fungus, is the main bioproduct used for insect control in productive areas worldwide [22].

The test subjects were kept at a temperature of 27 ± 2 °C, relative humidity of 80 ± 3%, and a 12-h photoperiod [12]. Sexed pairs collected and sanitized from field collections were placed in 500 mL plastic pots containing smaller pieces of cane billets (±10 cm), cut lengthwise (considered a natural diet) (Table 1). Each pair was separated into individual containers to ensure proper observation and management. The eggs obtained from oviposition on the food substrate were sanitized and monitored throughout the incubation period.

Containers intended for maintaining organisms in the laboratory were evaluated daily to ensure the ability to sustain the population under the described conditions. For experimental purposes, the animals were kept at room temperature and subsequently subjected to analysis. Since this is a non-lethal assessment method, the animals were returned to their original containers and marked in case it was necessary to reassess that individual.

### 2.4. Spectral Analysis

In total, larvae (three replicates), eggs (four replicates), pupae (four replicates), males (four replicates), and females (four replicates) were analyzed. The hyperspectral sensor Pika-L (Resonon Inc., Bozeman, MT, USA) [24] was used; this sensor has a spectral resolution of 2.1 nm, a spectral range of 400 to 1000 nm, and records approximately 281 spectral bands [24]. The data were processed to form a database using Spectronpro^®^ v. 3.0 software [24].

The sensor was securely mounted on a specific aluminum support designed exclusively for the Proximal SR benchtop and positioned 30 cm from the target. This support is sold as part of a complete system to ensure optimal alignment and functionality. Before the start of each evaluation session and during the procedure, calibration was performed using a radiometric calibration plate with a 99.9% reflectance reference for the ‘white’ calibration and was covered with a cap to obtain the ‘black’ reference (0% reflectance). The spectral profiles of all insects were obtained on black cardboard under the incidence of continuous spectrum light (tungsten halogen lamp).

**Figure 1:**
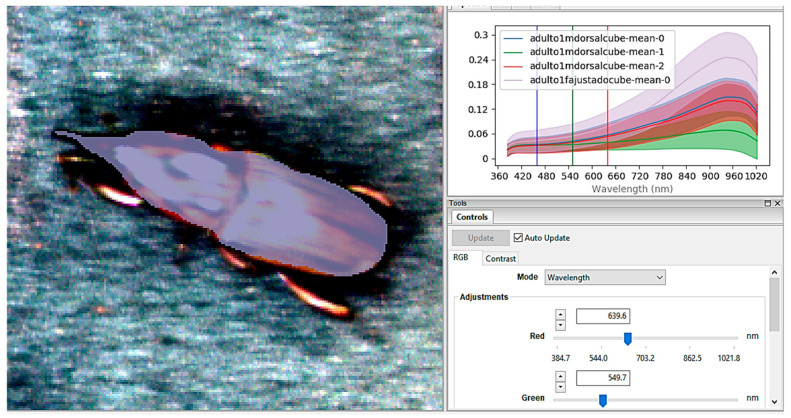
Acquisition of spectral data by the sensor and subsequent processing by the Spectronpro^®^ software [24], in the image of an adult *S. levis* (male).

Three individuals from each developmental stage, including three males and three females, were analyzed. From this, all developmental stages of *S. levis* (larva, pupa, adult, and egg) were evaluated, and their respective spectral signatures were determined from the images obtained (Figure 1).

For this study, the evaluated data was total, without the application of filters or pre- and post-processing smoothing methods for the spectral profiles. Generally, such methods are applied to highlight bands or continuous ranges that may be relevant, which is not the case in this study.

### 2.5. Statistical Analysis

Spectral data generates a large set of information, where each measured wavelength is considered a variable to be analyzed. This makes it difficult to group samples into different classes, especially when visual criteria are used, which tend to be less accurate [25]. Additionally, there is a high redundancy of information, as reflectance at the same wavelength shows a strong correlation [26]. Therefore, multivariate analyses were necessary to reduce the complexity of the data [27].

The data processing and analysis of the data matrix with wavelengths in the range of 400 to 1000 nm was performed using Python (v. 3.12), employing libraries such as Pandas, Matplotlib, and Seaborn for graphical visualization. Initially, the data were imported and organized into a Pandas DataFrame, which facilitated data manipulation and preprocessing. After importation, a portion of the data was discarded owing to the presence of noise, which is often observed at the edges of the spectral range. This step is crucial for ensuring the accuracy of the analyses. Using the cleaned data, specific spectral signatures were generated for each stage of the insect.

The reduced number of samples in our study can be attributed to the inherent complexity of hyperspectral datasets, which generate massive volumes of information. Each measured wavelength is treated as a variable to be analyzed, resulting in a high-dimensional space that makes it difficult to classify samples into different categories, especially when visual criteria are used, which can be less accurate than other methods.

Furthermore, the high redundancy of information in hyperspectral data, where reflectance at similar wavelengths is highly correlated, makes it challenging to implement analyses based on multiple repetitions of the same experiment. This situation makes it unfeasible to collect a high number of replicates, as an excess of data could compromise the efficiency of the analysis and lead to incorrect interpretation.

Consequently, we prioritized multivariate analyses to reduce data complexity and optimize the extraction of relevant information, which also required a controlled number of samples. We acknowledge that the small sample size may impact the statistical robustness of the results, and we suggest that future research consider strategies to increase the number of replicates and diversify the samples to enhance the conclusions regarding the spectral characterization of the organisms. To visualize these signatures, line graphs were created using Matplotlib, allowing for a clear comparison between the different stages. In addition, heatmaps were developed using the Seaborn library, providing a visual representation of spectral patterns and the variation in intensity across wavelengths.

In this way, an analysis of the spectral data of the insect *S. levis* was carried out using a visualization technique that combined a heatmap and a dendrogram using the Ward method [28]. This methodological choice is strategic because it provides a clear and intuitive way to explore large volumes of data and identify significant patterns. The Ward method is fundamental to this process, as it seeks to minimize the variance within each group when forming clusters [28]. This allows samples that share similar characteristics to be grouped together, aiding in the identification of developmental stages and the possible sexing of the samples, which are the central objectives of this study. Furthermore, this approach helps reduce the dimensionality of the data, which is essential in spectral analyses that often deal with large amounts of information. By eliminating redundancies, the heatmap and dendrogram make data interpretation more efficient and accessible.

The heatmap enables a graphical visualization of the relationships between different variables or samples, where color intensities represent similarities or dissimilarities. This makes reading the data easier, allowing researchers to quickly identify clusters and patterns without the need for complex statistical analysis. Thus, combining the heatmap with the Ward method not only optimizes the visualization of spectral data but also serves as a tool to achieve the study’s objectives: the creation of a spectral library dedicated to the life stages of *S. levis* and the analysis of their characteristics. This approach allows for a richer and more productive exploration of data, thereby facilitating the identification of relevant patterns.

## 3. Results

**Figure 2:**
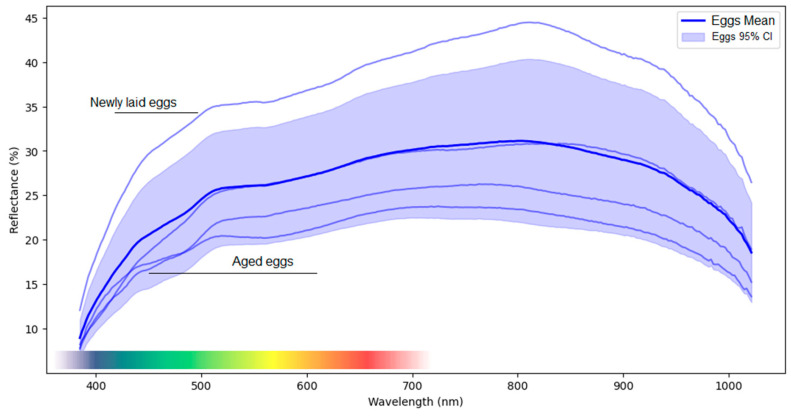
Mean spectral signature (solid dark blue line) and 95% confidence interval (shaded area) of *Sphenophorus levis* egg reflectance across the spectral range from 400 to 1000 nm. Lighter blue curves represent mean spectral profiles of eggs at different developmental times within the egg stage, illustrating the gradual spectral variation during egg development. The colored band at the bottom represents the portion of the spectrum visible to the human eye.

The spectral curve obtained for the eggs suggests that they (Figure 2) have a relatively high reflectance pattern in the visible (400–700 nm) and near-infrared (700–1000 nm) regions, which may be related to their chemical composition and surface structure.

The most recent eggs (1–2 days after being laid) show higher reflectance, which can be attributed to their fresher structure and the presence of internal fluids that have not yet degraded. This higher reflectance allows for better spectral distinction, making detection by imaging or spectroscopy methods easier. On the other hand, older eggs, more than 2 days after being laid, tend to become darker and more opaque as the inner membrane begins to dry out and the internal composition changes. This transition in reflectance can directly influence spectral detection and is correlated with the stages of egg maturation, contributing to the variation observed in confidence intervals (CIs). This suggests that egg age should be considered when interpreting spectral data and when assessing the health and viability of developing embryos.

**Figure 3:**
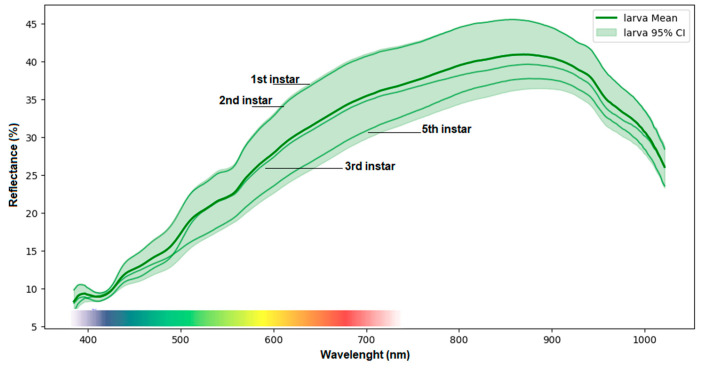
Mean Spectral Profile (solid dark green line) and Confidence Interval (CI) (95%) (shaded area) of the reflectance of *S. levis* larvae across the spectrum from 400 to 1000 nm. Colored bar indicates the spectrum that is visible to the human eye.

In contrast, the larvae showed a less dispersed pattern, with larvae at more advanced instar stages, indicating a decrease in reflectance throughout their development and a CI profile with less variation in the spectrum from 380 nm to 450 nm (Figure 3).

When the larvae were observed, the heterogeneous behavior persisted in the spectrum > 450 nm up to 1021 nm when compared to younger instars (“more reflective”), probably associated with physiological and nutritional changes, such as an increase in lipid deposition in the tissues in preparation for pupation.

**Figure 4:**
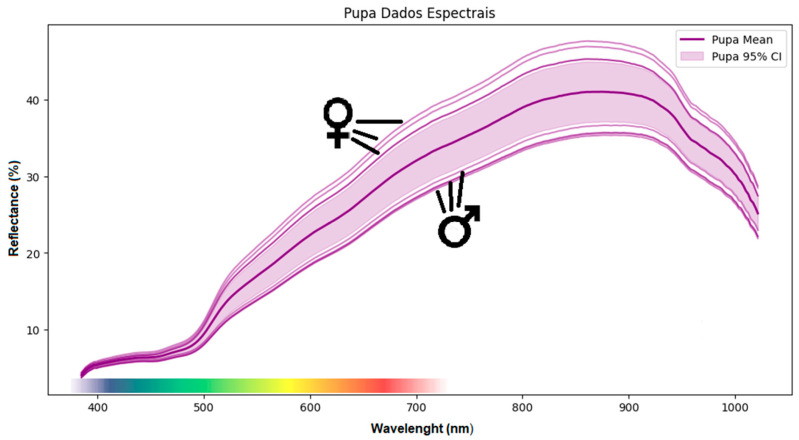
Mean spectral reflectance (solid purple lines) and 95% confidence intervals (shaded areas) of *Sphenophorus levis* pupae across the spectral range from 400 to 1000 nm. Separate mean spectral profiles are shown for female (♀) and male (♂) pupae to illustrate sex-related variation within the pupal stage. The colored band at the bottom represents the portion of the spectrum visible to the human eye.

Three days after their formation, the pupae presented a profile similar to that of males and females after the end of the pupal stage. Interestingly, the emerging adults could still be identified at this stage (Figure 4).

**Figure 5:**
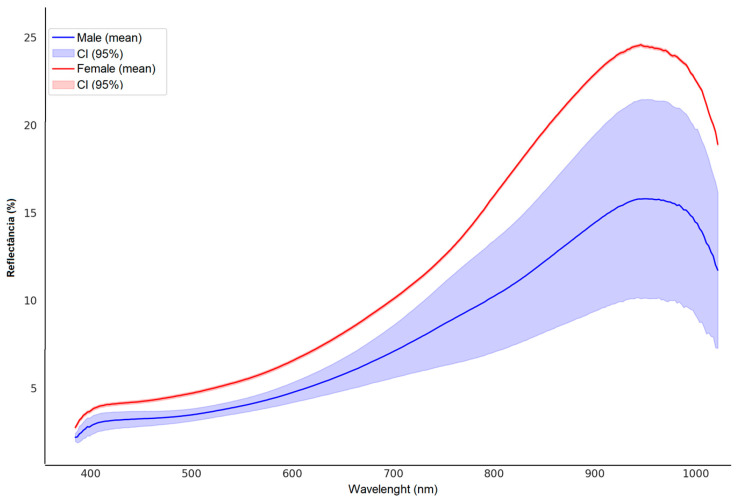
Mean spectral signature and 95% confidence interval of the reflectance of adult *S. levis* (blue: males and red: females) across the spectrum from 400 to 1000 nm.

In addition, it was possible to compare males and females, with the differentiation between males and females occurring primarily through their reflectance (%); that is, females have a more reflective body surface than males (Figure 5).

Graphically, males had a greater spread in the data, with a high variation in the CI, whereas females displayed a more homogeneous pattern. Checking the distance between the mean and CI for both groups allowed us to assume a statistical difference. This was confirmed through the analysis of variance of the mean (ANOVA), which indicated robust differences between the evaluated groups (Table 2).

ANOVA revealed a significant effect of developmental stage on reflectance (F_5,7194_ = 1073.18; *p* < 0.0001). The Tukey HSD test indicated significant differences between all stages (*p* < 0.05), with means ranging from 8.1% (adult males) to 31.6% (larvae). These results indicate that the optical spectrum clearly allows discrimination between the ontogenetic stages analyzed.

**Figure 6:**
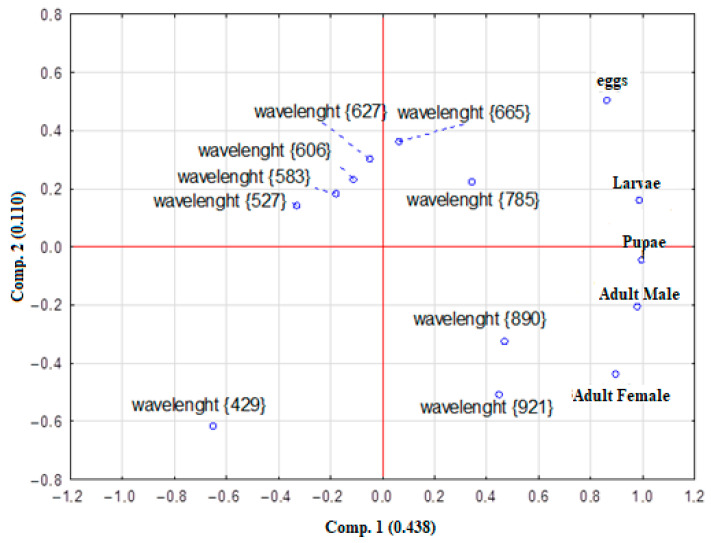
Principal Component Analysis (PCA).

Furthermore, to reduce data dimensionality and identify clustering patterns among the different developmental stages, PCA was performed, allowing for graphical visualization (Figure 6).

The PCA showed an explained variance for the first components (Comp.) 1 = 0.438% and Comp. 2 = 0.110%, considering biological data and that dimensionality reduction can lead to an increase in variance [29]; thus, the data can be considered robust [29]. The eigenvalues (eigenvectors) of the two components were greater than 1 and were retained according to Kaiser’s criterion [30], which states that an eigenvalue of 1 indicates that the component retains as much information as a single original variable.

Overall, there was a grouping of all insect life stages, with wavelengths displaying opposition to the insect life stages, probably indicating this. The PCA results indicated a grouping of males and females, with some proximity between two wavelengths (890 and 921 nm) in the near-infrared region; these wavelengths are likely important determinants of this insect life stage. The lower left region (429 nm) was isolated and may represent a wavelength with low variability in the spectra, possibly a specific characteristic point for all insect life stages.

**Figure 7:**
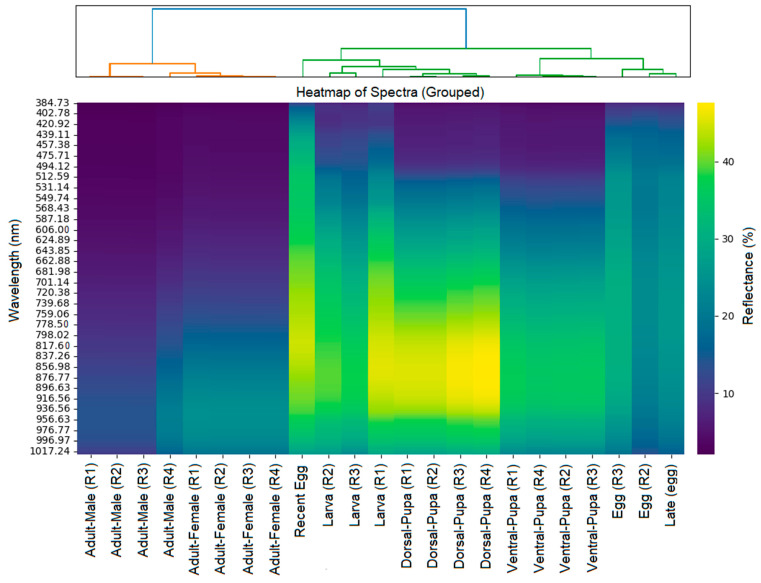
Heatmap of grouped spectra. Accompanied by a dendrogram (above) showing the clustering of different stages and developmental phases of *S. levis* variation. The vertical axis represents the wavelength (nm), with a color scale indicating the reflectance (%).

To assist with the overall visualization of the data, we conducted a hierarchical clustering analysis, the result of which was a dendrogram that positioned the insect life stages according to their reflectance indices (Figure 7).

The distribution of data was determined using hierarchical clustering analysis, where darker tones represent lower reflectance and lighter tones indicate higher reflectance. This suggests that larvae and pupae exhibit regions of high reflectance (yellow and green tones) at certain wavelengths, indicating structural or pigmentary differences in these more reflective stages at various wavelengths. Adults, both males and females, showed relatively uniform spectra with lower reflectance in specific spectral ranges. In fact, eggs appear to display patterns distinct from the other stages, possibly because of the structure of the shell and the chemical composition of the surface.

## 4. Discussion

The analysis of spectral data combined with an understanding of the biological cycle of *S. levis* suggests the critical importance of an integrated approach to pest management. Using the obtained data, we identified important information about the spectral signature of the different life stages of the insect. This highlights the need to integrate technologies, such as remote sensing, with biological knowledge of insects, thus opening new possibilities for biological studies, monitoring, and control of *S. levis*. Furthermore, this multidisciplinary approach not only supports the pillars of IPM but can also serve as a model for studying other important agricultural pests [14].

During insect development, the nutritional content of the eggs undergoes significant changes, reflecting the increasing nutritional demands of the developing embryo [31]. Insect eggs are primarily composed of proteins and lipids [31], with smaller amounts of carbohydrates, which are essential for providing resources for the development of the early stages. As the embryo develops, the stored nutrients are mobilized, and the proportion of lipids tends to decrease, whereas the consumption of proteins may increase, promoting growth and the formation of increasingly complex tissues, adapting to the specific needs of the different larval stages that follow hatching [32]. This pattern, generally observed in insects, highlights the complexity of stage transitions and the importance of an adequate nutritional composition from the egg stage to adulthood, thus ensuring the successful development and survival of populations [32].

For wavelengths relevant to eggs, a low reflectance zone was detected between 400 and 500 nm. This range coincides with the spectral behavior of antioxidant molecules and those related to protection against ultraviolet (UV) radiation [32,33] through the neutralization of reactive oxygen species (ROS) [33]. Carotenoids are common compounds in insect eggs [33] that possess high antioxidant power [33], and their presence may indicate a defense system against the host plant’s defenses [34]. When the egg is laid, in addition to the mechanical damage caused by the ovipositor, interactions mediated by metabolites from both the plants and insects begin [34]. The egg is exposed to an environment where the plant may activate localized defenses, for example, by increasing the production and release of reactive oxygen species (ROS) or phenolic compounds [34]. In this context, carotenoids help maintain the integrity of the embryo until hatching by neutralizing these free radicals.

However, in addition to nutritional changes, protecting embryos from environmental stresses, such as ultraviolet radiation and reactive oxygen species (ROS), is also important. For the wavelengths relevant to eggs, a zone of low reflectance was detected between 400 and 500 nm, coinciding with the spectral behavior of the antioxidant molecules [32,33]. Carotenoids, compounds that are very common in insect eggs, have high antioxidant power, and their presence may indicate a defense system against the host plant’s defenses [33,34]. When the egg is laid, in addition to the mechanical damage caused by the ovipositor, interactions mediated by metabolites from both the plants and insects begin [34]. The egg is exposed to an environment in which the plant can activate localized defenses, increasing the production and release of ROS or phenolic compounds [34]. In this context, carotenoids play a fundamental role in maintaining the integrity of the embryo until hatching by neutralizing free radicals.

When we observe the data in the spectral region within the near-infrared range (NIR: 700–1000 nm), the reflectance may be associated with low absorption and/or water content [35]. At this wavelength, it is possible for wavelengths in these bands to penetrate and provide information. This suggests that this developmental stage may be less dependent on external moisture for survival. However, when the environment is made less humid, mortality is observed in the egg stage, indicating that external moisture is crucial for egg viability (observational data). While eggs may possess mechanisms to endure low-humidity environments, excessive moisture can hinder gas exchange within the egg. This disruption ultimately poses a risk to embryonic development. Severe dehydration may compromise the integrity of the eggs and affect the viability of the developing embryos, highlighting the delicate balance required for optimal conditions.

For *Protophormia terraenovae* (Dip.: Caliphoridae), the authors conducted daily spectral measurements on different body sections of the pupae (anterior, middle, and posterior) [36]. The measurements showed that the posterior region provided the best distinction between the days of development, possibly associated with the dynamics of the posterior spiracles and the deposition of hydrocarbons on the cuticle. This suggests that significant chemical and physical changes are occurring in this region, associated with puparium formation and water loss [36,37], which have been occurring since the larval stage and are detectable in the final instars of the species [37].

In addition, there is empirical evidence that throughout the development of pupae, their spectral behavior resembles that of future adults, with observations of pupae showing both high and low reflectance. By evaluating these graphical differences, it is possible to a certain extent to determine the sex of males and females still in the pupal stage. As the pupae were evaluated at the same moment (3-day-old pupae), the differences were attributed to the metamorphosis process and the deposition of compounds for the development of future adults [36].

Regarding the larvae, variations in the spectra were detected for different instars, which can be attributed to increased cuticle deposition, pigmentation such as melanin, and the sclerotization of chitinous tissues. Early instar larvae (1st–2nd) tend to have thinner and less pigmented cuticles, which may result in lower reflectance in certain bands of the visible and/or near-infrared spectra. As they progress to later instars (3rd–5th), the cuticle may become thicker and more pigmented (for example, with melanin or other pigments) [38]. This is reflected in the change in the reflectance pattern observed in the curves [36].

Adults showed graphical variations in the evaluated spectral signatures, allowing for the sexing of males and females, including at the pupal stage. Additionally, it can be stated that females exhibit low variability in the spectral signature that reflects their body surface, whereas males show a wide variation in the data cloud. Although the PCA and the dendrogram with the heat map indicated a very close clustering between males and females, the spectra allowed for an efficient graphical distinction between the two groups.

The PCA and heatmap associated with the dendrogram indicate a grouping of pupae and larvae (upper right quadrant), suggesting that they share similar optical properties, possibly associated with less sclerotized cuticle, associated with spectral bands between 600 and 785 nm. In contrast, adults have distinct spectra, indicating changes in the cuticle structure and chemical composition of the surface. The data for adults seem more correlated to spectral bands in the near-infrared range (>890 nm), which may be associated with changes in cuticle density and structure.

The insect cuticle is a complex structure composed mainly of chitin, structural proteins, lipids, and other pigments. This chemical composition directly influences the cuticle’s interaction with different wavelengths of light, making it detectable using spectral analysis techniques. Different spectral bands can be used to identify variations in the cuticle structure, detect changes in insect development, and assess the presence of pathogens or environmental stresses.

### 4.1. Ecological Implications and Integrated Pest Management

The separation of adults (males vs. females) may reflect adaptive strategies related to some type of visual communication (unlikely but possible), thermoregulation, and/or camouflage within their habitat. In contrast, the pupae and larvae displayed similar spectra, suggesting less spectral differentiation at these intermediate developmental stages, which may be linked to a phase of reduced exposure to light or predators, or even that their natural enemies may employ non-visual strategies (olfactory, tactile, and/or chemical).

Recently, harvestman populations were discovered in sugarcane fields in Ribeirão Preto (São Paulo, Brazil), where *Liogonyleptoides inermis* Mello-Leitão, 1922 (Opiliones: Gonyleptidae) was the most dominant species, associated with traps baited with live *S. levis* specimens [39]. The authors suggested a possible olfactory/chemical location capability followed by tactile foraging by *L. inermis* on *S. levis* [39]. Generalist predators that make little use of visual resources to identify prey [40] include species that display visual predation behavior, such as *Heteromitobates discolor* (Arachnida: Opiliones) [40].

Defensive behaviors are essential for living organisms to survive in their environment, and thanatosis and spectral variation may be related to the capabilities of adults. For example, a pattern of low reflectance in adults may indicate adaptation to shaded environments [41], which is indeed the case in this study [6,9]. Considering the peaks of nocturnal activity in adults [9], the high reflectance in the near-infrared (NIR) range may indicate thermal camouflage strategies, helping to avoid predators that use infrared-sensitive vision [42]. This may also be related to thermal regulation [42]. As solar radiation is absent at night, the ambient temperature drops, and organisms may avoid excessive heat loss by reflecting infrared radiation.

Furthermore, the width of the Confidence Interval (CI) observed in males may reflect the variable selective pressures between males and females. Males display a heterogeneous pattern of body surfaces, whereas females exhibit a homogeneous pattern. It can be assumed that males are exposed to greater risks when releasing pheromones to identify more suitable oviposition sites for females to lay their eggs. However, females have a higher average displacement than males, averaging 3 m day^−1^ and reaching up to 5 m day^−1^ [43]. These data suggest that females are more exposed, which creates an apparent contradiction.

This contradiction observed between the behavioral dynamics of males and females suggests that different reproductive strategies are at play, as well as ecological variables that may influence this dynamic. One possible hypothesis to explain this difference is that males, having a heterogeneous body surface pattern, may use this variation as a camouflage strategy or as a form of distinct signaling in complex environments, making them less visible to predators while actively searching for females. In contrast, the greater average movement of females may be associated with a more careful habitat selection pattern, where the search for suitable sites for oviposition involves a trade-off between safety and environmental quality.

This raises the hypothesis that females may be more selective regarding habitat type, requiring more movement to find locations with ideal conditions, such as the presence of high-quality food resources and/or less intraspecific competition. Thus, the hypothesis that can be raised is that both males and females are under different selective pressures that shape their behavior. While males may have developed adaptations to maximize their visibility and attractiveness, taking risks in pursuit of mating and/or specific aggregation sites, females may prioritize safety and the quality of oviposition sites, which require more strategic movement. This balance between attraction and safety may be crucial for the reproductive success of both sexes in the population dynamics of the species. Further investigations should examine these patterns in different environments and contexts and assess how morphological variations influence interactions between individuals and their efficiency in reproduction.

Genetic differences between male and female insects can be attributed to the distinct selective pressures stemming from their different lifestyles [44]. These differences are often the result of natural and/or sexual selection, which influences gene expression differently between sexes [45]. Gene expression and selective pressure in many insects lead to phenotypic differences between males and females, mediated by differential gene expression, in which genes are expressed at different levels in each sex [46]. This can lead to a “sexual conflict,” where sexually antagonistic selection favors alleles that are beneficial to one sex but detrimental to the other [47].

Thus, the study of the spectral profiles of the developmental stages of *S. levis* makes a significant contribution to the understanding of the biology and ecology of this pest. The analysis of spectral data revealed crucial information about the spectral characteristics at different stages of the insect’s life cycle, allowing for differentiation between larvae, pupae, and adults (males and females) based on their specific spectral profiles. These findings are particularly relevant for integrated pest management (IPM), an approach that seeks to use combined control methods to minimize economic damage caused by agricultural pests.

Accurate identification of the life stages of *S. levis* through remote sensing can facilitate faster and more effective interventions, enable the use of automated equipment for insect rearing, and allow farmers to implement control strategies before damage becomes economically significant.

### 4.2. Study Limitations

The study on the spectral signatures of different developmental stages of *S. levis* yielded promising results and established a useful foundation for identifying life stages and sexing insects, following established protocols and standards used in previous research on other insect species. However, the limitations of this study should be considered, as they may introduce generalizations that call for further reflection and investigation into variations in the biology and ecology of *S. levis*.

First, sampling was conducted in a single geographic area, which may not reflect the genetic and ecological variability in *S. levis* populations from different regions. Environmental factors, such as climate, soil type, and the presence of predators, can influence both the spectral characteristics and behavior of insects. Therefore, including individuals from different regions in the analyses could broaden our understanding of the spectral and behavioral responses of insects under various ecological conditions.

Furthermore, although this study identified differences in spectral signatures between sexes, the behavioral implications of these differences were not addressed. Aspects such as mate-searching behavior, territoriality, and reproductive strategies may influence the spectral characteristics observed of the animals. A more detailed analysis of insect behavior could provide valuable context for interpreting the spectral data obtained.

Additionally, it is worth noting that the evaluated insect exhibits peculiar characteristics that are scarcely documented in scientific literature. This limitation may lead to some generalizations regarding its behavior and ecology, but it also tends to drive new studies and research aimed at deepening the understanding of the insect’s biology and its interactions with the environment.

This study focuses on a single insect species as a model system. Further studies involving multiple species are necessary to assess the broader applicability of the method, as well as to refine our understanding of the spectral data collected. These limitations highlight the importance of exercising caution when interpreting the results and suggest directions for future studies. Additional research is essential to consolidate knowledge about the biology and management of *S. levis* by adopting a more comprehensive and integrative approach that considers the complexities of the insect and its environment.

## 5. Conclusions

The data obtained can be used to improve the sexing of males and females, determine egg development time, and categorize larval instar. The integration of multiple spectral ranges provides a more robust approach to understanding the developmental dynamics and interactions of insects with their habitat. The ability to accurately differentiate developmental stages represents a significant advantage over traditional techniques, allowing entomology professionals to make faster and more precise estimates, with direct implications for understanding population dynamics and developing laboratory breeding systems, including the potential for automation.

Notably, this study is pioneering, as it initiates the creation of a spectral library of *S. levis* for all developmental stages, which contributes to the knowledge of various aspects of the biology and ecology of this pest insect. Furthermore, these data can be used to improve strategies for the identification, management, and control of this species. The ability to accurately differentiate developmental stages using non-destructive methods presents a significant advantage over traditional techniques.

## Figures and Tables

**Table 1:** Chemical composition of the natural diet of *Sphenophorus levis* based on the literature [23].

Natural Diet [23]	(% Dry Matter)
Dry Matter	27.87
Moisture Content	72.13
Moisture Content Crude Protein	2.32
Acid Detergent Insoluble Protein	2.38
Neutral Detergent Insoluble Fiber Corrected for Ash and Protein	47.68
Insoluble Fiber in Acid Detergent	44.78
Ether Extract	1.41
Total Carbohydrates	94.27
Neutral Detergent Soluble Carbohydrates	43.58
Mineral Matter	2.00

**Table 2:** Total mean reflectance and standard deviation of the different developmental stages of *S. levis*.

Development Stage	Mean Reflectance and Standard Deviation (%)				
Adult Male	8.10 ± 0.13 e				
Adult Female	12.18 ± 0.21 d				
Pupae	22.63 ± 0.27 c				
Egg	27.32 ± 0.25 b				
Larva	31.56 ± 0.37 a				
Effects	SS	dr	MS	F	*p*
Intercept	3,310,210	1	3,310,210	34,077.31	0.00
Development stage	521,235	5	104,247	1073.18	0.00
Error	698,813	7194	97		

Different letters in the same column indicate statistically significant differences according to Tukey’s test (*p*-value < 0.05).

## Data Availability

The data presented in this study are available on request from the corresponding author. The data are not publicly available due to a confidentiality agreement regarding the collection site of the organisms, established with the partner company involved in the research.

## Abbreviations

The following abbreviations are used in this manuscript:

ROS	Reactive Oxygen Species
CI	Confidence Interval
IPM	Integrated Pest Management
EDL	Economic Damage Level
NIR	Near-Infrared
nm	Nanometers
RS	Remote Sensing
UV	Ultraviolet
